# Supplementary figures and images for: AdipoRon, adiponectin receptor agonist, improves vascular function in the mesenteric arteries of type 2 diabetic mice
Source: PLoS One. 2020 Mar 17;15(3):e0230227. doi: 10.1371/journal.pone.0230227 (PMC7077821; doi:10.1371/journal.pone.0230227)

Fig. 3A

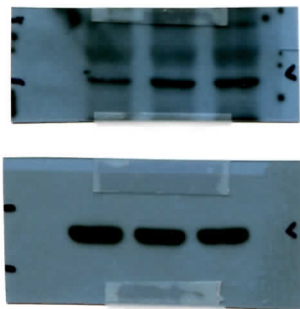

Fig. 3B

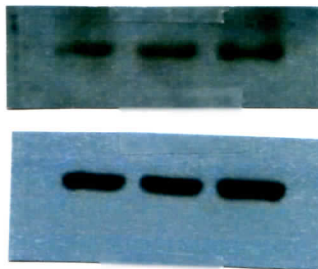

Fig. 3C

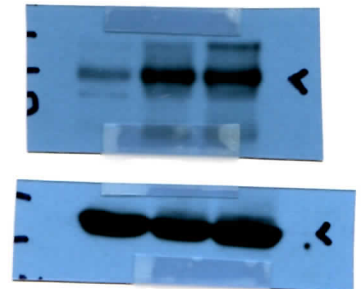

Fig. 3D

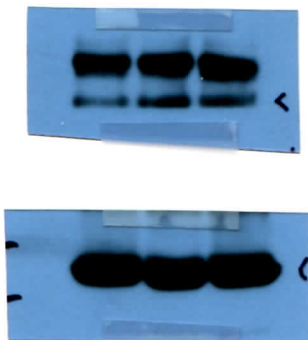

Fig. 4A

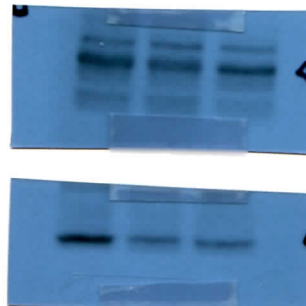

Fig. 4B

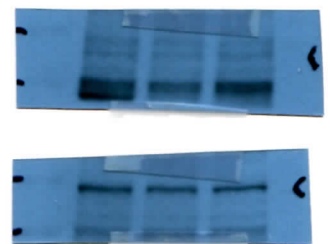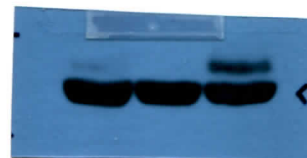

Fig. 5A

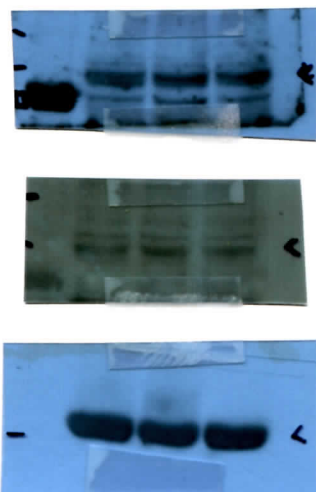

Fig. 5B

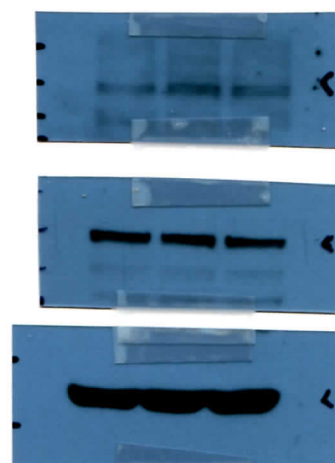

Supplement: S1 Fig — (PDF) [file pone.0230227.s001.pdf]
