# Supplementary material for: AdipoRon, adiponectin receptor agonist, improves vascular function in the mesenteric arteries of type 2 diabetic mice
Source: PLoS One. 2020 Mar 17;15(3):e0230227. doi: 10.1371/journal.pone.0230227 (PMC7077821; doi:10.1371/journal.pone.0230227)
Supplement: Attachment — Submitted filename: Responses to reviewer_20200128.docx [file pone.0230227.s002.docx]

**RE: PONE-D-19-29078
MS TITLE: AdipoRon, adiponectin receptor agonist, improves vascular function in the mesenteric arteries of type 2 diabetic mice**

We thank the Editor and Reviewers for their careful review and do appreciate the constructive comments that strengthen our manuscript. The manuscript was revised in accordance with the suggestions of the reviewers.

**List of the Changes:**

Page 1: The word count and number of references were changed.

Page 1,7: An author was added.

Page 5-7: Introduction section and Method section were changed.

Page 11-12: Result for cultured smooth muscle cells was added.

Page 15: Discussion section was changed.

Page 18: References were added.

Page 21: Figure legend section was changed.

Figure 5: Data for Western blot analysis with cultured smooth muscle cells were added.

**Response to Reviewers:**

**Reviewer #1:**

The author Dr Choi and the colleagues demonstrated that AdipoRon improves vascular function in the mesenteric arteries in diabetic mice model via an endothelium independent manner and activated MLCP by MYPT1 could be the reason for this induced effect. Although multiple observations and experiments were made, it is lack of convincing evidence to support the current conclusion, robust mechanism is needed as well.

**Comment 1**: AdipoRon is an adiponectin receptor activator and it has been proved that mesenteric arteries expressed AdiRs. The unchanged the protein levels does not equal to unchanged activity. Nitric oxide blocker or adiponectin receptor specific knockout model need to be applied to solidate the finding.

***Answer****: We agree with the reviewer. We thought it is helpful to use adiponectin receptor specific knockout model in this study, however, it is hard to obtain the knock out model in our laboratory. We also agree that unchanged level of AdiRs means unchanged signaling of the receptor. That’s is why we observed the APPLs in the same tissues. In the revise manuscript, we added the data showing direct effect of AdipoRon in the cultured cells. We hope it could consolidate our findings.*

***Comment 2****:* The evidence to support the finding that demonstrate adipoRon can directly activate MYPT of MLCP in the mesenteric arteries need to further obtain evidence from cultured cells. Meanwhile, why is the MYPT is the signals AdipoRon relied on? Cause-effect approaches is required to enforce the current conclusion.

***Answer****: We thank the review for raising this concern. Following the reviewer’s comment, we added the data showing that AdipoRon reduced phosphorylation level of MYPT1 in cultured arterial smooth muscle cells in the revised manuscript (Please see page 11 and figure 5B). The reason we thought AdipoRon affects MYPT1 signal is following. 1) AdipoRon treatment normalized enhanced myogenic responses. 2) AdipoRon treatment did not affect eNOS phosphorylation. From these results, we assumed that AdipoRon could affect Ca^2+^ sensitization mechanism. Thus we observed CPI-17 and MYPT1 level and found AdipoRon reduced phosphorylation level of MYPT1 and CPI-17 phopshorylation was not affected. (Data not shown).*

**Reviewer #2:**

The authors of the present study have examined the vasorelaxant effects of AdipoRon, an adiponectin receptor agonist, using mesenteric arteries from type 2 diabetic mice. Although the observed findings appear to be interesting, there are concerns regarding the novelty of the present study as noted below.

Introduction:
***Comment 1****:*The Introduction section is very weak. The first two paragraphs are descriptive, and they appear to be a review of the previously known facts about obesity, diabetes, and adiponectin receptor signaling.

***Answer****: We thank the reviewer for raising this concern. We agree with the reviewer’s comment. We improved the introduction section followed by the reviewer’s comment. (Please see introduction section. )*

***Comment 2****:* The very first article on AdipoRon, an adiponectin receptor agonist, was published in 2013. In the third paragraph of Introduction, the authors have cited only this article by Okada-Iwabu (Nature 2013).

***Answer****: We improved the introduction section followed by the reviewer’s comment. (Please see page 5.)*

***Comment 3****:* From the Introduction section, it is clear that the present manuscript lacks novelty. Previously, it has been reported that AdipoRon induces vasorelaxation by mechanisms independent of endothelium-dependent relaxing factors and AMPK activation (Hong et al. Microcirculation 23, 207-220, 2016).

***Answer****: In the previous study, Hong et al. showed that AdipoRon induced vasorelaxation without a marked decrease in VSMC [Ca^2+^]_i_ in various arteries. However, they showed the acute effect of AdipoRon on the vessels in normal animals and did not suggest exact mechanism that AdipoRon dilates the arteries. In the present study we showed that chronic, in vivo treatment of AdipoRon induced improvement of vascular function in the type 2 diabetic mice. Furthermore, we suggested that AdipoRon treatment increased the phospho-AMPK and decreased MYPT1 phosphorylation in diabetic mice while there was no change in the level of eNOS phosphorylation.*

Methods and Results:
***Comment 1****:* Using pressure and wire myography, Hong et al have previously reported the vasorelaxant effects of AdipoRon in different vascular beds, including skeletal muscle, cerebral, coronary and mesenteric arteries. Thus, the findings of the present study are not novel (Microcirculation 23, 207-220, 2016).

***Answer****: It would be the similar answers for the comment 1. In the previous study, Hong et al. showed that AdipoRon induced vasorelaxation without a marked decrease in VSMC [Ca^2+^]_i_ in various arteries. However, they showed the acute effect of AdipoRon on the vessels in normal animals and did not suggest exact mechanism that AdipoRon dilates the arteries. In the present study we showed that chronic, in vivo treatment of AdipoRon induced improvement of vascular function in the type 2 diabetic mice. Furthermore, we suggested that AdipoRon treatment increased the phospho-AMPK and decreased MYPT1 phosphorylation in diabetic mice while there was no change in the level of eNOS phosphorylation.*

***Comment 2****:* For the present study using type 2 diabetic db^−^/db^−^ mice, it is unclear as to why the authors have used db^−^/db^+^ mice as the control group instead of the wild-type mice.

***Answer****: The db^−^/db^+^ mice represent the heterozygous counterpart of diabetic db/db mice and are carriers of the mutated gene of the leptin receptor but do not become diabetic at any stage during their lifespan. Thus we consider the db^−^/db^+^ as control mice for db^−^/db^−^ mice.*
